# Feeding Practices during the COVID-19 Lockdown: Is there a Difference between Stay-at-Home Mothers and Work-from-Home Mothers?

**DOI:** 10.21315/mjms2022.29.6.16

**Published:** 2022-12-22

**Authors:** Seok Tyug Tan, Darshni Saundara Rajan

**Affiliations:** Faculty of Health and Life Sciences, Management and Science University, University Drive, Selangor, Malaysia

**Keywords:** feeding practices, working mothers, stay-at-home mothers, COVID-19, lockdown

## Abstract

This study aims to compare the feeding practices adopted by stay-at-home mothers and work-from-home mothers during the COVID-19 lockdown. Feeding practices were assessed using a 39-item validated Comprehensive Feeding Practices Questionnaire (CFPQ). The Multivariate Analysis of Covariance (MANCOVA) was used to determine the mean difference in feeding practices by maternal employment status after adjusting for the mother’s age and educational attainment and the child’s age and gender. The current study revealed that a noticeable proportion of mothers adopted feeding practices that encourage balance and variety (4.45 ± 0.62), promote a healthy environment (4.34 ± 0.81) and impose dietary restrictions for health (4.32 ± 1.04) during the COVID-19 pandemic. There was no significant difference (*P* > 0.05) in the mean scores of feeding practices across maternal employment status, except for the emotion regulation and modelling subscales. Work-from-home mothers had a significantly higher mean score in the emotion regulation subscale than stay-at-home mothers (*F* = 14.892, *P* < 0.001). In comparison to work-from-home mothers, stay-at-home mothers had a significantly higher mean score in the modelling subscale during the COVID-19 pandemic (*F* = 4.163, *P* = 0.044). In conclusion, there was just a modest variation in the feeding practices adopted by stay-at-home mothers and work-from-home mothers in this unprecedented pandemic.

## Introduction

The Federal Government of Malaysia has imposed three nationwide lockdowns or Movement Control Orders (MCOs) to curb the spread of COVID-19. The first two lockdowns took effect from 18 March 2020 to 3 May 2020 (MCO 1.0) and 13 January 2021 to 4 February 2021 (MCO 2.0), in response to an increase in the daily confirmed cases across the country (1). As of 28 September 2021, Malaysia is in the third nationwide lockdown, with more than 222,000 people infected by the virus (2).

Several restrictions were applied during MCO enforcement; for instance, all learning institutions were ordered to close due to the difficulty of maintaining social distancing and physical attendance at the workplace was only permitted at a limited capacity (3, 4). These social-distancing restrictions resulted in more time spent at home, and parents now have complete control over their child’s eating behaviour and food intake (5). Therefore, the current study aims to compare the feeding practices of stay-at-home mothers with work-from-home mothers following the re-imposition of MCO (MCO 2.0).

## Methods

### Study Design and Population

Data collection was conducted from 13 January 2021 through 4 February 2021 (approximately 10 months after MCO 1.0 and during the enforcement of MCO 2.0). Ethical approval was obtained from the Research Ethics Committee of Management and Science University. Informed consent was acquired from mothers prior to answering the first survey questions.

Mothers with at least one child aged between 2 years old–11 years old and able to read English were recruited using a combination of purposive and snowball sampling techniques. On the other hand, mothers who were laid off as a result of the COVID-19 outbreak or those who needed to be physically present at their workplaces were excluded from this study. An anonymous web-based questionnaire was hosted on Google Forms and circulated to mothers through social-media platforms such as Facebook, Instagram, WhatsApp, Twitter and TikTok. The sample size was determined using the G*Power software version 3.1 (Heinrich-Heine-Universität Düsseldorf, Düsseldorf, Germany) with an 80% power at α = 0.05 to detect a medium effect size of 0.25 (6). Hence, a minimum sample size of 36 mothers is required for this study.

### Socio-Demographic Characteristics

Socio-demographic information, including the mother’s age, educational attainment, employment status before and during the COVID-19 pandemic, and the child’s gender and age were reported by mothers. A stay-at-home mother is an unemployed mother who stays at home to take care of their children, whilst a work-from-home mother is a working mother who needs to work remotely during the pandemic lockdown (7).

### Feeding Practices During the COVID-19 Pandemic

A 39-item validated Comprehensive Feeding Practices Questionnaire (CFPQ) was used to assess maternal feeding practices during the home confinement (8). This 39-item were then further categorised into 12 feeding practice subscales, including encourage balance and variety (3-item), healthy environment (2-item), restriction for health (2-item), teaching about nutrition (2-item), modelling (4-item), monitoring (4-item), food as reward (2-item), pressure to eat (3-item), involvement (3-item), child control (4-item), emotion regulation (3-item) and restriction for weight control (7-item). The operational definitions for all feeding practices are as described by Raymond Leprince et al. (9).

### Statistical Analysis

Data analysis was conducted using SPSS Statistics for Windows, version 26.0 (IBM Corp., Armonk, NY, USA). When applicable, descriptive statistics including frequency, percentage, mean and standard deviation (SD) were used to describe socio-demographic characteristics and maternal feeding practices. The Multivariate Analysis of Covariance (MANCOVA) with the Bonferroni post-hoc test was applied to determine the mean difference in feeding practices according to maternal employment status (stay-at-home mothers versus work-from-home mothers). The mother’s age, educational attainment and, the child’s age and gender were treated as covariates in the MANCOVA analysis. A *P*-value of less than 0.05 (*P* < 0.05) was statistically significant.

## Results

Table 1 summarises the socio-demographic characteristics of the respondents. This cross-sectional study enrolled 22 stay-at-home mothers and 100 work-from-home mothers of children aged 2 years old–11 years old. The majority of mothers aged 30 years old and above (75.4%), are tertiary educated (50.8%), with a daughter (54.1%) and/or a child aged 2 years old–6 years old (53.3%). The mean age of mothers was 33.44 ± 4.52 years old.

**Figure 1:**
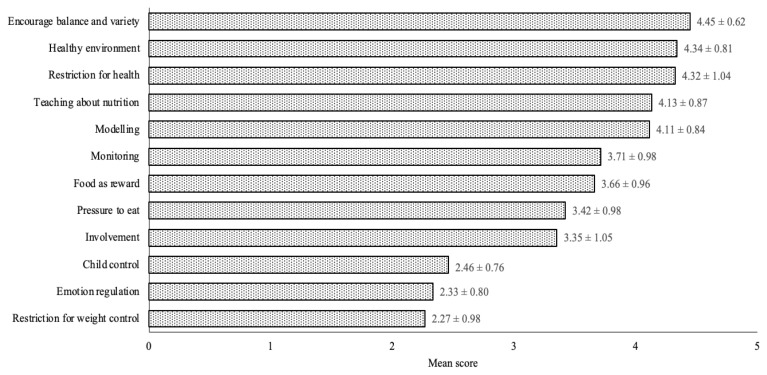
Maternal feeding practices during the MCO 2.0

Figure 1 shows the maternal feeding practices during the MCO 2.0. A noticeable proportion of mothers adopted feeding practices that encourage balance and variety (4.45 ± 0.62), promote a healthy environment (4.34 ± 0.81) and impose dietary restrictions for health (4.32 ± 1.04) during the COVID-19 pandemic. Conversely, child control (2.46 ± 0.76), emotion regulation (2.33 ± 0.80) and restriction for weight control (2.27 ± 0.98) were the least preferred feeding practices in the pandemic.

Table 2 indicates the mean difference in feeding practices by maternal employment status during the COVID-19 pandemic. Emerging findings indicated that feeding practices were significantly dependent on maternal employment status during the pandemic lockdown (*F* = 5.036, *P* < 0.001, Hotelling’s T^2^ = 0.576, partial η^2^= 0.365). The work-from-home mothers had a significantly higher mean score in the emotion regulation subscale than stay-at-home mothers (*F* = 14.892, *P* < 0.001). In contrast, stay-at-home mothers had a significantly higher mean score in the modelling subscale than work-from-home mothers (F = 4.163, *P* = 0.044).

## Discussion

Encourage balance and variety, healthy environment and restriction for health were the top three feeding practices adopted by mothers in Malaysia during the COVID-19 home confinement. These top-ranked feeding practices were not-so-surprising, given the fact that having a proper and healthy diet in this unprecedented pandemic is crucial for a robust immune system (10). Emerging findings also suggested that the majority of mothers encouraged healthy eating behaviour in their children by providing nutritious options at home while enforcing specific dietary restrictions to achieve optimal health during the pandemic. To a certain extent, these practices are generally in adherence to practical advice on maintaining a healthy diet by the World Health Organization (11).

The current study demonstrated that work-from-home mothers attained a significantly higher score in the emotion regulation subscale but a significantly lower mean score in the modelling than stay-at-home mothers. Mothers face enormous challenges as a result of the COVID-19 lockdown, for example, work-from-home mothers are at risk of pandemic burnout due to the strain of juggling distant work, childcare and managing additional household chores during the pandemic (12). This may partially rationalise the usage of comfort foods by work-from-home mothers to alleviate negative emotional arousals such as boredom and temper tantrums in children during the pandemic. In addition, working mothers are also reported to have less time bonding with their children, fewer family dinners and less time spent on food preparation than part-time and unemployed mothers (13). Emerging findings suggested that needing to work remotely during the COVID-19 lockdown made parental role modelling of healthy eating less impossible for work-from-home mothers.

This study adopted a web-based approach due to face-to-face data collection was not feasible during the country lockdown. Therefore, one of the limitations naturally includes not reaching those without access to the internet. In view of the small sample size in this study, the emerging findings may not accurately reflect the feeding practices of all mothers in Malaysia. Additionally, future research should evaluate the effect of household composition, wealth and access to cooking facilities on parental feeding practices (14). Despite those previously mentioned, the current study was the first to ascertain the feeding practices adopted by mothers during the pandemic lockdown.

## Conclusion

In a nutshell, mothers in Malaysia recognised the critical nature of healthy eating habits during the pandemic. There was just a modest variation in the feeding practices adopted by stay-at-home mothers and work-from-home mothers in this unprecedented pandemic.

## Figures and Tables

**Table 1:** Socio-demographic characteristics of mothers and children

Characteristic	Frequency *n* (%)	Mean ± SD
Child		
Gender		
Boy	56 (45.9)	-
Girl	66 (54.1)	
Age (years old)		
2–6	65 (53.3)	6.21 ± 2.93
7–11	57 (46.7)	
Mother		
Age (years old)		
≤ 30	30 (24.6)	33.44 ± 4.52
> 30	92 (75.4)	
Educational attainment		
Secondary and below	60 (49.2)	-
Tertiary	62 (50.8)	
Employment status		
Stay-at-home mothers	22 (18.0)	
Work-from-home mothers	100 (82.0)	-

**Table 2:** Feeding practices by the maternal employment status during the COVID-19 pandemic

Feeding practice^1^	Stay-at-home mothers	Work-from-home mothers	*F*-value	*P*-value

	Mean ± SD	Mean ± SD		
Encourage balance and variety	4.44 ± 0.65	4.45 ± 0.62	0.121	0.729
Healthy environment	4.36 ± 0.71	4.34 ± 0.84	0.002	0.965
Restriction for health	4.25 ± 0.80	4.34 ± 1.08	0.295	0.588
Teaching about nutrition	4.11 ± 0.83	4.14 ± 0.89	0.615	0.435
Modelling	4.45 ± 0.69	4.03 ± 0.86	**4.163**	**0.044** *
Monitoring	3.73 ± 1.08	3.71 ± 0.96	0.010	0.919
Food as reward	3.52 ± 1.17	3.69 ± 0.91	0.310	0.579
Pressure to eat	3.44 ± 0.92	3.42 ± 1.00	0.006	0.936
Involvement	3.65 ± 1.32	3.29 ± 0.98	2.106	0.149
Child control	2.69 ± 0.90	2.41 ± 0.73	2.463	0.119
Emotion regulation	1.76 ± 0.85	2.46 ± 0.73	**14.892**	**< 0.001** *
Restriction for weight control	2.52 ± 1.31	2.21 ± 0.90	2.073	0.153

Notes:

1 Covariates: mother’s age, educational attainment and, child’s age and gender;

* Mean difference was tested using MANCOVA with Bonferroni post hoc test. The significant difference was considered at *P* < 0.05

## References

[1] Tan ST, Tan CX, Tan SS (2021). Physical activity, sedentary behavior, and weight status of university students during the COVID-19 lockdown: a cross-national comparative study. Int J Environ Res Public Health.

[2] Ministry of Health Malaysia (2021). Situasi terkini COVID-19 di Malaysia sehingga.

[3] Tan ST, Tan SS, Tan CX (2021). Screen time-based sedentary behaviour, eating regulation and weight status of university students during the COVID-19 lockdown. Nutr Food Sci.

[4] National Security Council SOP perintah kawalan pengerakan (PKP).

[5] Joseph Louis SP, Tan ST (2021). Socio-demographic disparities in the eating behaviour of Malaysian children during the COVID-19 lockdown. Osong Public Heal Res Perspect.

[6] Cohen J (1988). Statistical power analysis for the behavioral sciences.

[7] Vyas L, Butakhieo N (2021). The impact of working from home during COVID-19 on work and life domains: an exploratory study on Hong Kong. Policy Des Pract.

[8] Shohaimi S, Wei WY, Zalilah MS (2014). Confirmatory factor analysis of the malay version comprehensive feeding practices questionnaire tested among mothers of primary school children in Malaysia. Sci World J.

[9] Raymond Leprince J, Sarina S, Rahmah Begam BM (2020). Parental child feeding practices and growth status of Orang Asli children in Negeri Sembilan, Malaysia. Br Food J.

[10] Aman F, Masood S (2020). How nutrition can help to fight against COVID-19 pandemic?. Pakistan J Med Sci.

[11] World Health Organization (WHO) Healthy diet.

[12] Clark S, McGrane A, Boyle N, Joksimovic N, Burke L, Rock N (2021). “You’re a teacher you’re a mother, you’re a worker”: gender inequality during COVID-19 in Ireland. Gender Work Organ.

[13] Bauer KW, Hearst MO, Escoto K, Berge JM, Neumark-Sztainer D (2012). Parental employment and work-family stress: associations with family food environments. Soc Sci Med.

[14] Kabir A, Maitrot MRL (2017). Factors influencing feeding practices of extreme poor infants and young children in families of working mothers in Dhaka slums: a qualitative study. PLoS ONE.

